# Supplementary material for: Visual Features in the Perception of Liquids
Source: Curr Biol. 2018 Feb 5;28(3):452–458.e4. doi: 10.1016/j.cub.2017.12.037 (PMC5807092; doi:10.1016/j.cub.2017.12.037)
Supplement: Document S1. Figures S1–S4 and Table S1 [file mmc1.pdf]

Current Biology, Volume *28*

Supplemental Information

Visual Features in the Perception of Liquids

Jan Jaap R. van Assen, Pascal Barla, and Roland W. Fleming

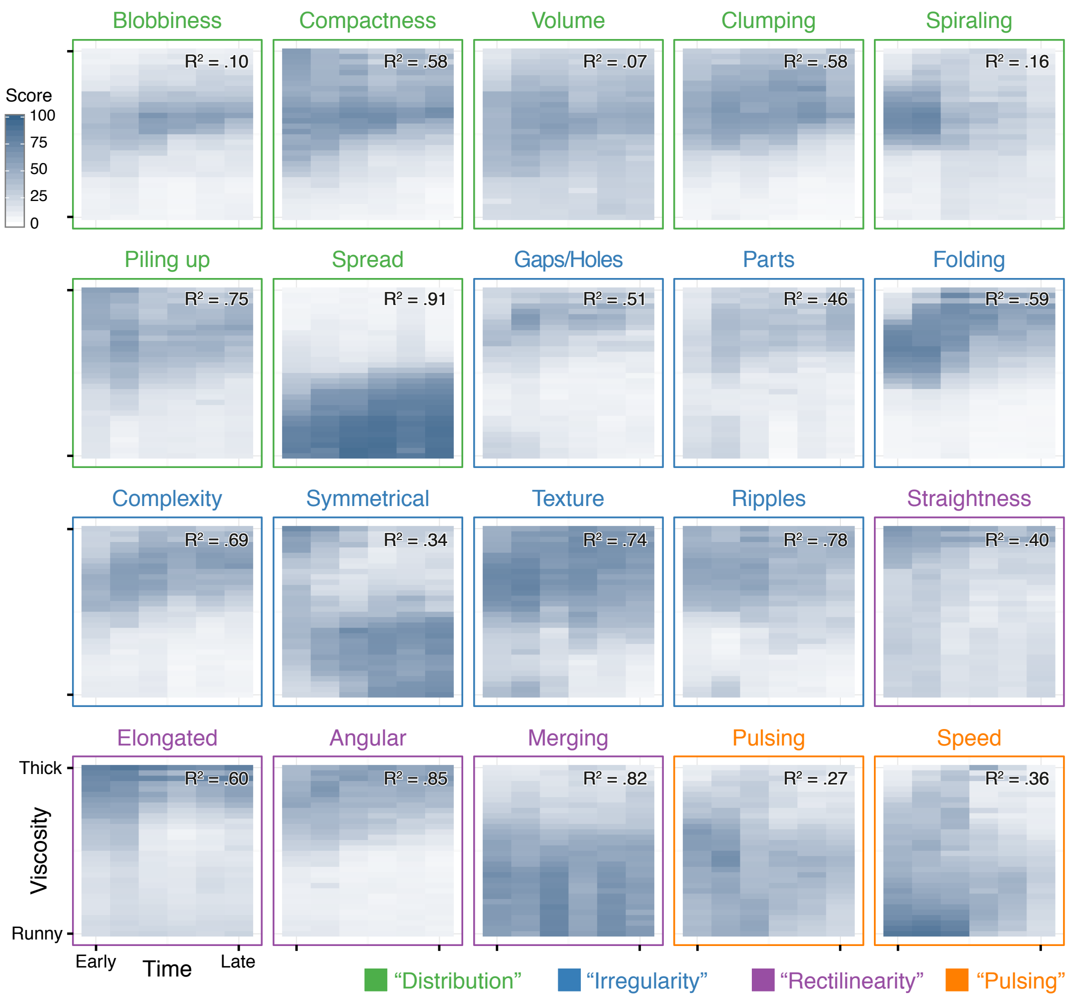

Figure S1: Experiment 3 feature ratings. Related to Figure 1: Mean midlevel feature ratings of Experiment 3 for all twenty shape features, colour coded by the factors for which they have the largest weights. Y-axis: viscosity (32 viscosities, from runny 0.001 Pa·s to thick 80.30 Pa·s); X-axis: time (six time periods of 1.67 seconds or 10 seconds divided by six). The feature judgments generally varied in complex (often non-monotonic) ways as a function of viscosity and time period.

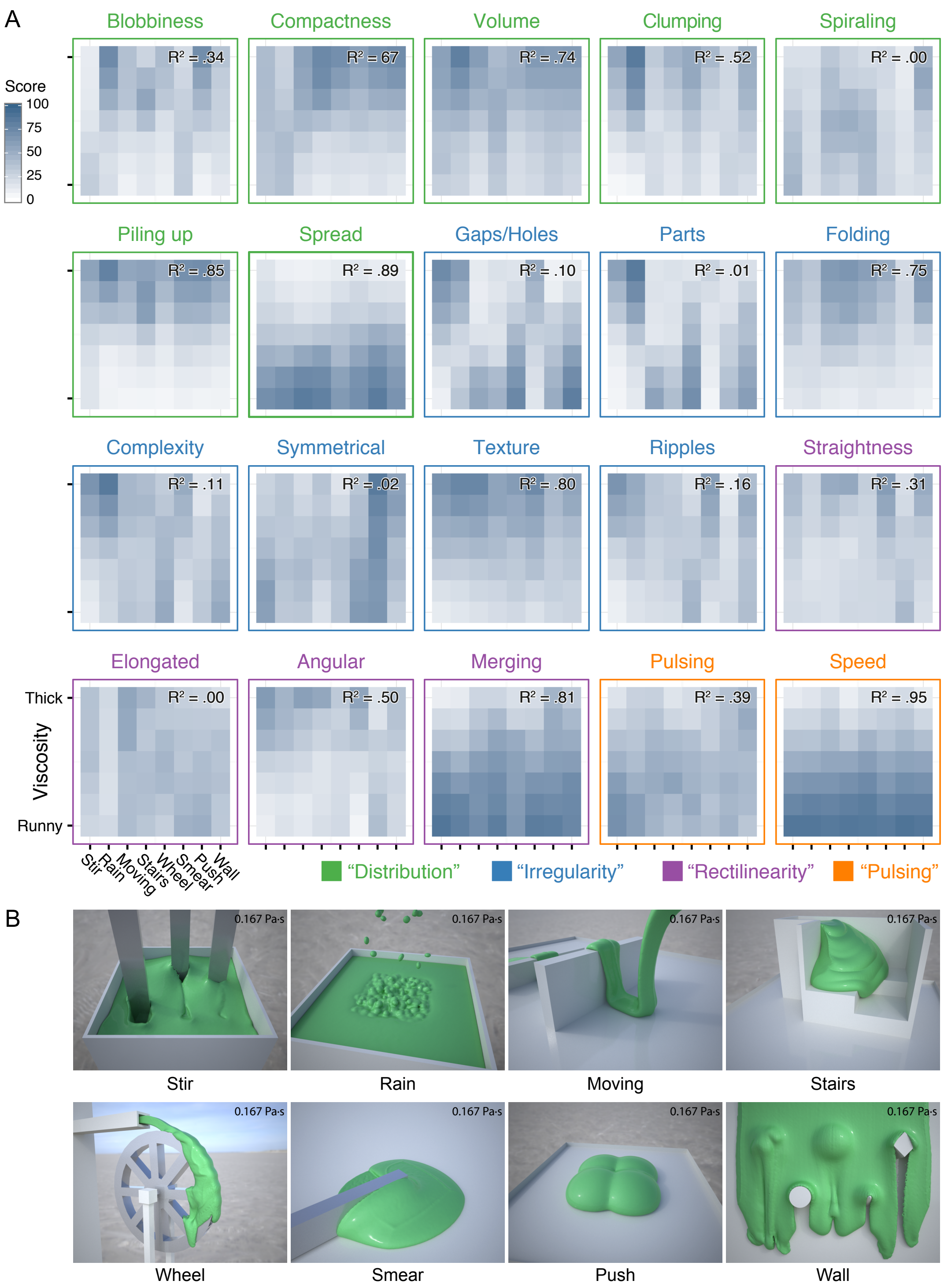

Figure S2: Experiment 4 feature ratings. Related to Figure 2: A) Mean midlevel feature ratings of Experiment 4 for all twenty shape features, colour coded by the factors for which they have the largest weights. Y-axis: viscosity (7 viscosities, from runny 0.004 Pa·s to thick 7.74 Pa·s); X-axis: eight different scenes. The feature judgements vary considerably on scene-by-scene basis, indicating that our simulated scenes capture a wide range of different liquid behaviours. B) The eight scenes with an intermediate viscosity (0.167 Pa·s).

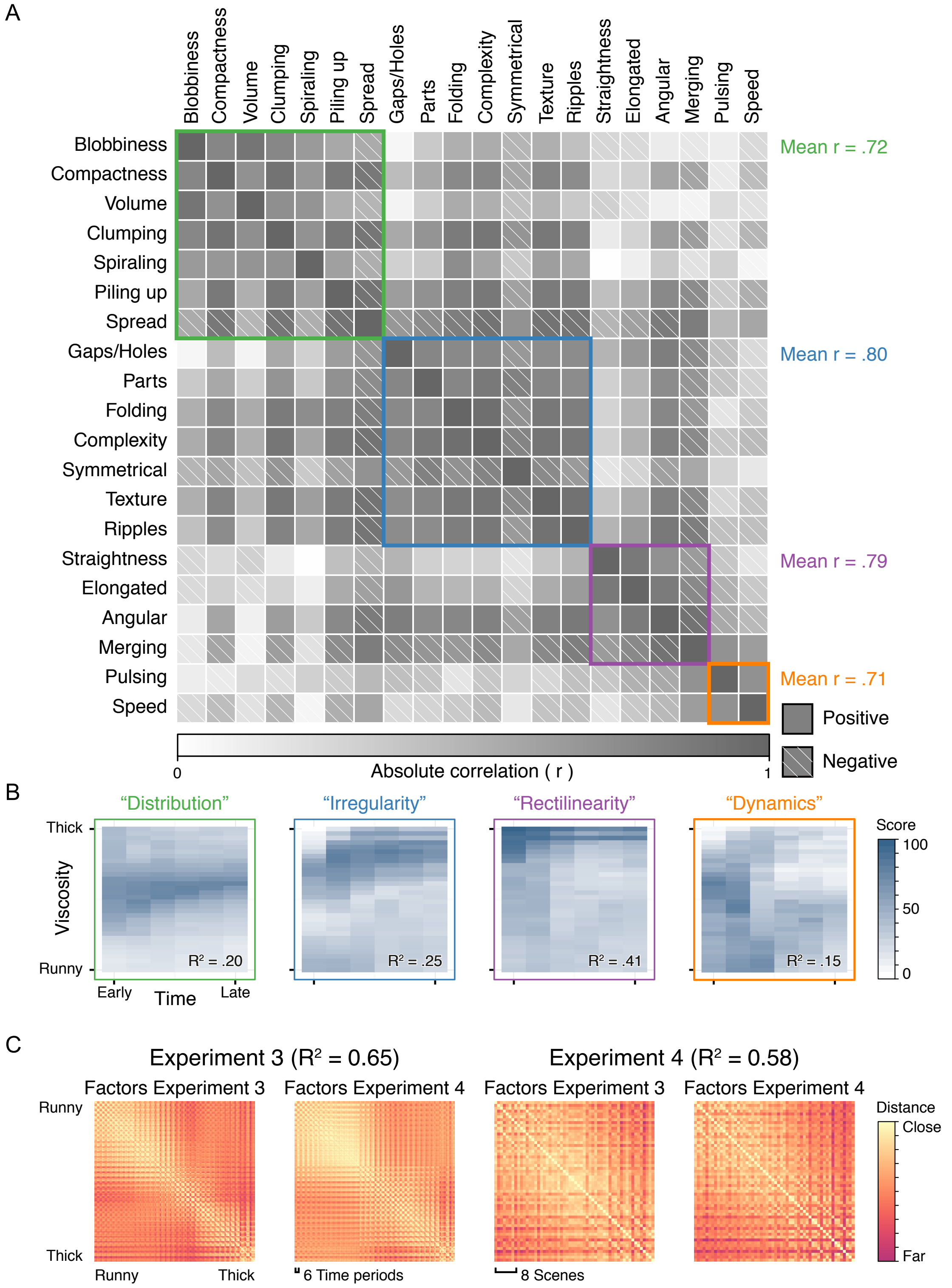

Figure S3: Factor analysis. Related to Figure 3: A) Correlations between perceptual feature ratings from Experiment 3; gray shade indicates absolute magnitude of correlation, stripes indicate negative correlations. Coloured frames indicate the four factors; *r*-values indicate mean absolute correlations between features within each factor, the overall mean absolute correlation is *r* = 0.57. B) The four factors that result from applying the factor loadings to the twenty perceptual features. R^2 values indicate linear regression between each

factor on its own and the viscosity ratings from Experiment 1. The complementary nature of the factors means that each on its own predicts only a small proportion of the variance, but combined in a multiple linear regression, they explain 97% of the variance in the viscosity ratings. C) Representational Dissimilarity Matrices (RDMs) for the two regression models, derived from the Pouring scene (left) and the 8 scenes (right). In each case we apply factors derived from Experiment 3 and Experiment 4 and quantify how similar these factor spaces are. Colours represent the Euclidean distance between the corresponding pair of stimuli in the respective factor-space representation (red: dissimilar, yellow: similar). The R^2-score indicates the explained variance between the lower triangles of the two matrices (i.e., diagonal and upper triangle excluded from analysis).

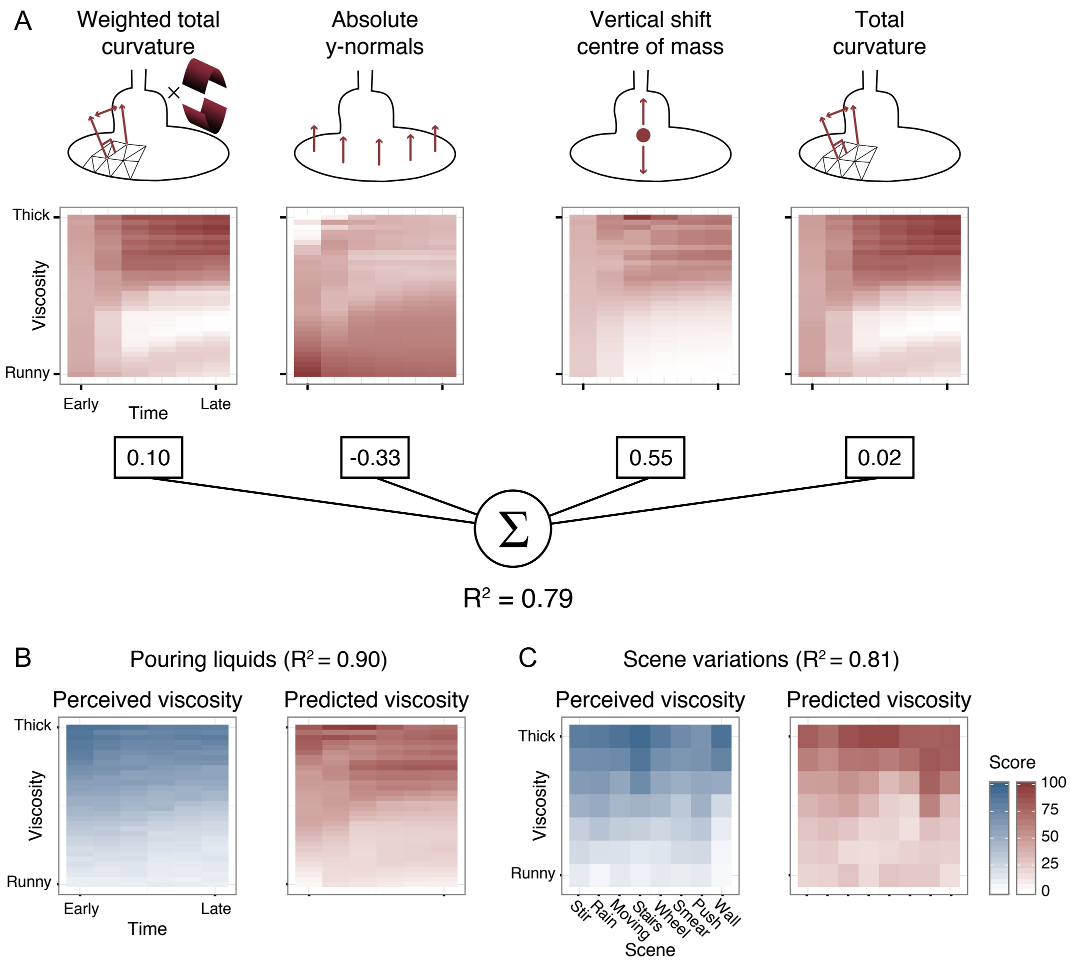

Figure S4: 3D measurements. Related to Figure 3D: A) Schematics of the four mesh measurements used in the 3D stimulus-computable model, along with values for all stimuli in the pouring scene (Experiments 1 and 3). A regression model using the weights below each mesh measurement predicts 79% of the variance in viscosity ratings across all scenes. B) Human viscosity ratings (blue) and mesh model predictions (mauve) for Experiment 1 (pouring liquids). R^2 score indicates regression for a model fit only to these data. C) Human viscosity ratings (blue) and mesh measurements predictions (mauve) for Experiment 3 (eight scenes). R^2 score indicates regression for a model fit only to these data.

Shape feature	Description
Symmetrical	How symmetrical the shape is
Compactness	How tightly arranged the shape is
Clumping	How much the shape features distinct clumps
Folding	How much does the shape fold back on itself
Pulsing	How much does shape change in a rhythmical repeating way
Merging	How the shape absorbs into itself
Speed	How quickly the shape changes or moves
Spiraling	How much does the shape change in a spiral movement or form
Elongated	How much is the shape stretched out in a single direction
Texture	How much does the surface have variations rather than being smooth
Blobbiness	How rounded or bulbous is the shape
Piling up	How piled up is the shape
Straightness	How much does the shape contain straight features
Complexity	How complex is the shape i.e. not simple
Spread	How spread out is the shape
Ripples	How much does the shape feature ripples
Gaps/Holes	How much does the shape shows gaps or holes
Parts	How much does the shape consist of multiple parts rather than one single part
Angular	How sharp or angular are the features of the shape
Volume	How voluminous is the shape

Table S1: Feature list. Related to Figure 1 and 2: First column showing the twenty different shape features used in Experiment 3 and 4. Second column showing the corresponding description given as additional information to make it easier to rate the corresponding feature (here translated from German). The separation in the middle of the table shows the two different feature groups, each observer only rated ten features out one of these two groups.
