# Supplementary material for: Visual Features in the Perception of Liquids
Source: Curr Biol. 2018 Feb 5;28(3):452–458.e4. doi: 10.1016/j.cub.2017.12.037 (PMC5807092; doi:10.1016/j.cub.2017.12.037)
Supplement: Document S2. Article plus Supplemental Information [file mmc4.pdf]

Report

Current Biology

Visual Features in the Perception of Liquids

Highlights

- Observers are remarkably good at visually inferring the viscosity of flowing fluids
- They use multiple midlevel shape and motion features to do so
- Four factors predict perceived viscosity constancy surprisingly well
- The features take wildly divergent stimuli and organize them by viscosity

Authors

Jan Jaap R. van Assen, Pascal Barla, Roland W. Fleming

Correspondence

mail@janjaap.info (J.J.R.v.A.), pascal.barla@inria.fr (P.B.), roland.w.fleming@psychol.uni-giessen.de (R.W.F.)

In Brief

van Assen et al. use the visual perception of flowing liquids to uncover the computations underlying the brain's sensory inferences about materials. By comparing observers' viscosity ratings with perceived shape features, they show how the brain exploits 3D shape and motion cues to infer viscosity across contexts despite dramatic image changes.

van Assen et al., 2018, Current Biology *28*, 452–458
February 5, 2018 © 2018 The Authors. Published by Elsevier Ltd.
https://doi.org/10.1016/j.cub.2017.12.037

CellPress

Current Biology
Report

Visual Features in the Perception of Liquids

Jan Jaap R. van Assen,[1,4,*] Pascal Barla,[2,*] and Roland W. Fleming[3,*]
[1]Department of Experimental Psychology, University of Giessen, Otto-Behaghel-Strasse 10F, 35394 Giessen, Germany
[2]Inria Bordeaux Sud-Ouest, 200 Av de la Vieille Tour, 33405 Talence Cedex, France
[3]Department of Experimental Psychology, University of Giessen, Otto-Behaghel-Strasse 10F, 35394 Giessen, Germany
[4]Lead Contact
*Correspondence: mail@janjaap.info (J.J.R.v.A.), pascal.barla@inria.fr (P.B.), roland.w.fleming@psychol.uni-giessen.de (R.W.F.)
https://doi.org/10.1016/j.cub.2017.12.037

SUMMARY

Perceptual constancy—identifying surfaces and objects across large image changes—remains an important challenge for visual neuroscience [1–8]. Liquids are particularly challenging because they respond to external forces in complex, highly variable ways, presenting an enormous range of images to the visual system. To achieve constancy, the brain must perform a causal inference [9–11] that disentangles the liquid's viscosity from external factors—like gravity and object interactions—that also affect the liquid's behavior. Here, we tested whether the visual system estimates viscosity using "midlevel" features [12–14] that respond more to viscosity than other factors. Observers reported the perceived viscosity of simulated liquids ranging from water to molten glass exhibiting diverse behaviors (e.g., pouring, stirring). A separate group of observers rated the same animations for 20 midlevel 3D shape and motion features. Applying factor analysis to the feature ratings reveals that a weighted combination of four underlying factors (distribution, irregularity, rectilinearity, and dynamics) predicted perceived viscosity very well across this wide range of contexts (R^2 = 0.93). Interestingly, observers unknowingly ordered their midlevel judgments according to the one common factor across contexts: variation in viscosity. Principal component analysis reveals that across the features, the first component lines up almost perfectly with the viscosity (R^2 = 0.96). Our findings demonstrate that the visual system achieves constancy by representing stimuli in a multidimensional feature space—based on complementary, midlevel features—which successfully cluster very different stimuli together and tease similar stimuli apart, so that viscosity can be read out easily.

RESULTS AND DISCUSSION

If the estimation of viscosity proceeds hierarchically—through a weighted combination of midlevel features describing dynamic 3D shape properties—it should be possible to identify such features and use them to predict perceived viscosity across variations in other scene variables. To test this hypothesis, we simulated liquids with a wide range of viscosities interacting with a variety of different scenes (see Movies S1 and S2). In Experiment 1 we made detailed measurements of viscosity perception in a simple scene in which each liquid poured vertically onto an object on a plane (Figure 1A). The 10 s animations, depicting liquids with 32 different viscosities, were divided into six (1.67 s) time periods. On each trial, observers viewed eight videos of liquids with different viscosities from the same time period and rated the perceived viscosity by adjusting sliders for each video. Results are shown in Figure 1B. Consistent with previous work [14–16], we find that observers are excellent at judging viscosity: the regression between their ratings and physical truth was R^2 = 0.96, $F(1,190)$ = 4,941, $p < 0.001$. There was also a mild tendency to see later time periods as runnier. The range of responses across observers is shown in Figure 1E.

Comparing viscosities across liquids is relatively straightforward if all other scene factors are held constant. The deeper challenge is to achieve constancy—i.e., generalization across contexts. To investigate constancy, in Experiment 2 we created a series of scenes in which liquids underwent qualitatively different behaviors, such as oozing through holes, being stirred in a container, or interacting with a waterwheel (Figure 2A; Movie S1). Seven viscosities were simulated, and observers again rated viscosity, this time for the entire 10 s of each animation (see STAR Methods for details). We found a significant decline in viscosity constancy across scenes, as indicated by the different rates at which the columns in Figure 2B change from light to dark. Nevertheless, observers were still very well able to differentiate and order the seven simulated viscosities across qualitatively different behaviors, yielding a regression between the ratings and physical truth of R^2 = 0.92, $F(1,54)$ = 656.7, $p < 0.001$. The range of responses across different individuals is shown in Figure 2E.

Next, we sought to identify a set of midlevel shape and motion cues that predict viscosity perception. Rather than identifying potential cues through physical analysis, we took a data-driven approach in which we selected a broad set of hypotheses through phenomenology, which could then be tested, rejected, and refined through experimentation. To do this, we viewed the "pouring liquids" stimulus set and brainstormed features that (1) described aspects of the stimuli's 3D shape and motion, (2) varied across stimuli, and (3) could be described to participants verbally. We also asked four naive observers to brainstorm a list of features describing the liquids. Although the terms they identified were not identical to ours, they were judged by another group of observers to overlap substantially with our list,

 © 2018 The Authors. Published by Elsevier Ltd.
This is an open access article under the CC BY license (http://creativecommons.org/licenses/by/4.0/).

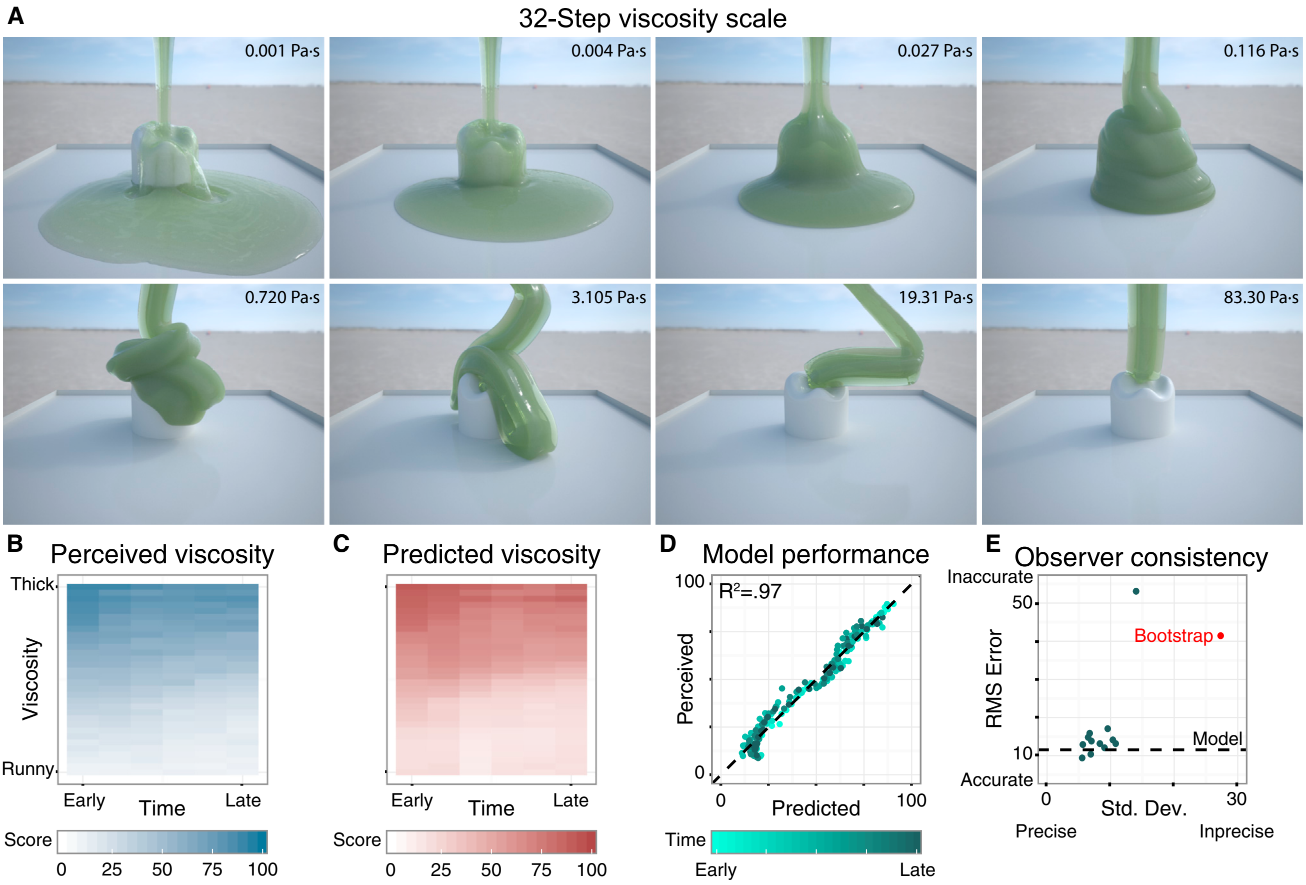

Figure 1. Experiment 1 Model Predictions

(A) Eight equally spaced viscosity stimuli spanning the full range of viscosities (frame 90 of 300).

(B) Mean viscosity ratings for all videos in Experiment 1.

(C) Predicted viscosity for same stimuli, based on the four-factor model.

(D) Scatterplot comparing model predictions to mean responses across observers and repetitions. Darker greens indicate later time periods.

(E) Root mean square errors relative to ground truth viscosities and standard deviation of responses across repetitions for each observer (dots); red dot indicates bootstrapped estimate of random performance based on 1,000 random draws.

See also Figure S1, Movies S1 and S2, and Table S1.

suggesting that we had identified a reasonable set of features to test. Importantly, we view the initial feature list as a superset of potential cues—i.e., hypotheses—which we sought to cull through subsequent analyses.

To do this, in Experiments 3 and 4, two new groups of observers viewed the same videos as in Experiments 1 and 2, but instead of rating viscosity, they rated the 20 features (e.g., compactness, elongation, pulsing, clumping; see Table S1 for a complete list with specific instructions). None of the features referred to the liquids' material properties. Instead, they targeted the stimulus's 3D shape and motion characteristics to test the hypothesis that viscosity is inferred from specific weighted combinations of such cues.

Results for three of these features with pouring liquids (Experiment 3) are shown in Figure 3A (see Figure S1 for all 20 features). Unlike viscosity ratings, the feature judgments often varied in complex, non-monotonic ways as a function of viscosity and time period. This means the different features provide potentially complementary cues about the liquid. Although some individual features predict viscosity perception in some scenes, few features predict all the data well on their own. Instead, the brain likely combines multiple cues to achieve more robust estimates of viscosity. There were strong correlations between features (Figure S3A), suggesting a smaller number of true underlying factors describing the liquids' shape and motion.

To test this, we performed a factor analysis (Figure 3). A Horn test [17] revealed four basic factors (each a weighted combination of the feature ratings; Figure S3): (1) distribution—describing the extent the liquid clumped together versus spread out; (2) irregularity—describing how complex and detailed its shape was; (3) rectilinearity—capturing how straight and angular the liquid appeared; and (4) dynamics—describing its motion properties. When a new group of nine observers were asked to judge these factors directly, the responses correlated significantly (mean R^2 = 0.52) with the factors derived from the feature ratings on the same stimuli. The process of applying factor analysis allowed us to narrow down the broader list of 20 features to four more refined and specific hypotheses.

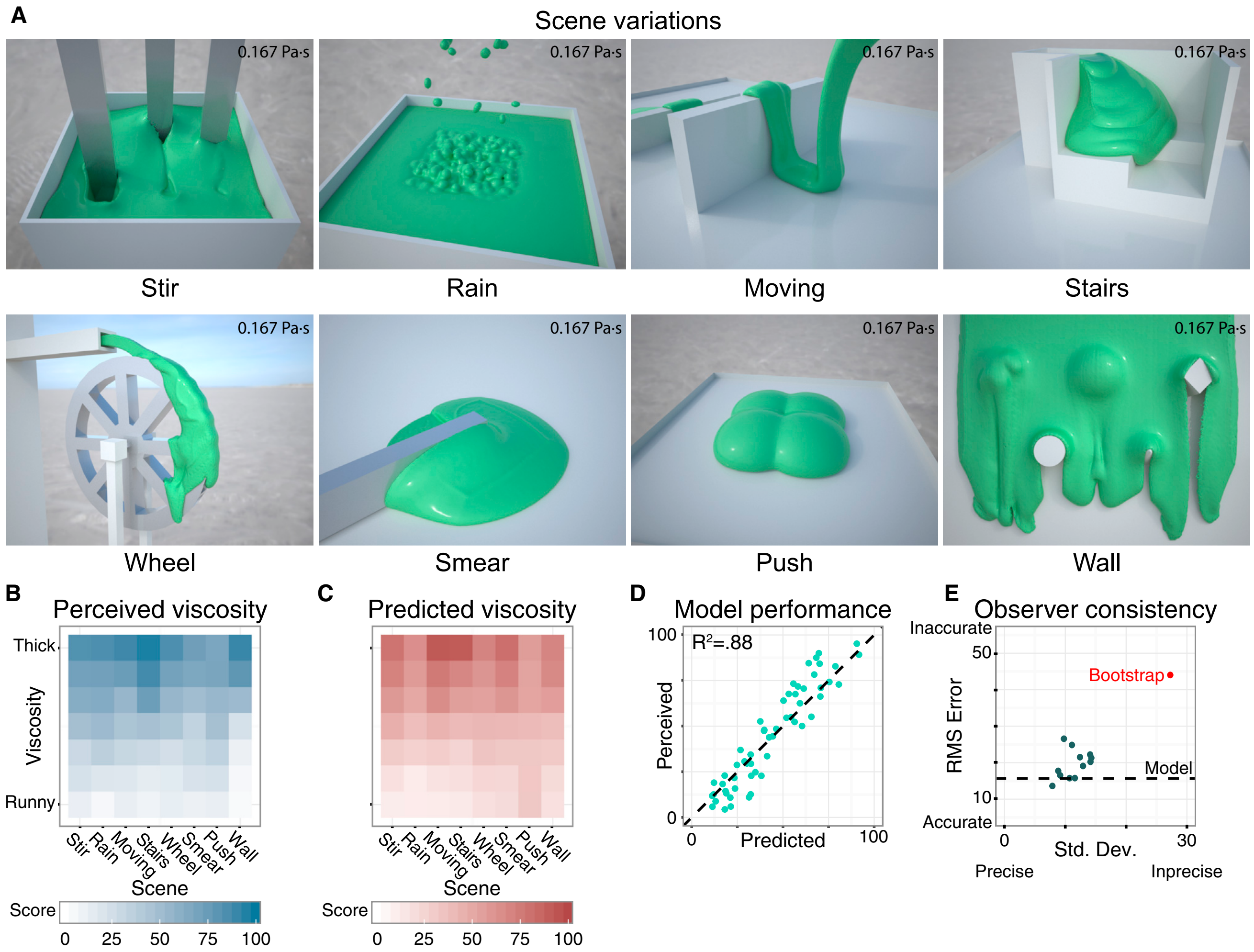

Figure 2. Experiment 2 Model Predictions

(A) Eight different scenes simulated with the same viscosity of 0.167 Pa·s.

(B) Mean viscosity ratings for all scenes in Experiment 2.

(C) Predicted viscosity for same stimuli, based on the four-factor model.

(D) Scatterplot comparing model predictions to mean responses across participants and repetitions (from [B] and [C]).

(E) Root mean square errors relative to ground truth viscosities and standard deviation of responses across repetitions for each observer (dots); red dot indicates bootstrapped estimate of random performance based on 1,000 random draws.

See also Figure S2, Movies S1 and S2, and Table S1.

To test whether these factors could predict viscosity perception, we performed a multiple linear regression, using the factors derived from Experiment 3 to predict the viscosity ratings data from Experiment 1. The model predicts the viscosity data extremely well, $R^2 = 0.97$, $F(4,187) = 1,386$, $p < 0.001$, far better than random predictors (a bootstrapping analysis with 1,000 repetitions revealed 185 predictors would be required to achieve equivalent non-significant performance). This indicates that a simple weighted linear combination of dynamic 3D shape features is sufficient to explain perceived viscosity. Note, again, that the combination of factor analysis and regression allows us to reduce our initial hypotheses and to quantify the relative roles of individual cues. Of course, on its own, our finding does not strictly imply that the estimation of midlevel features is prior to the inference of viscosity. It is logically possible that observers derived their feature judgments from the perceived viscosity. However, we suggest that the detailed—often non-monotonic—feature ratings makes this unlikely. On grounds of parsimony, it seems more likely that viscosity is inferred from the midlevel features than vice versa.

The key challenge of viscosity perception is to achieve constancy across dramatic changes in the liquid's behavior. To test how well the model predicts viscosity constancy, we applied the factor loadings and regression weights derived solely from the "pouring" scene (Experiments 1 and 3) to the feature ratings from Experiment 4 to measure how well the model predicted viscosity perception in the other eight scenes (Experiment 2). Results are shown in Figure 2D. Despite having no new training data or additional free parameters, the model generalizes to the eight new scenes remarkably well, ($R^2 = 0.88$, $F(1,54) = 391.4$, $p < 0.001$). These results confirm that a relatively small number of midlevel stimulus characteristics—related to how

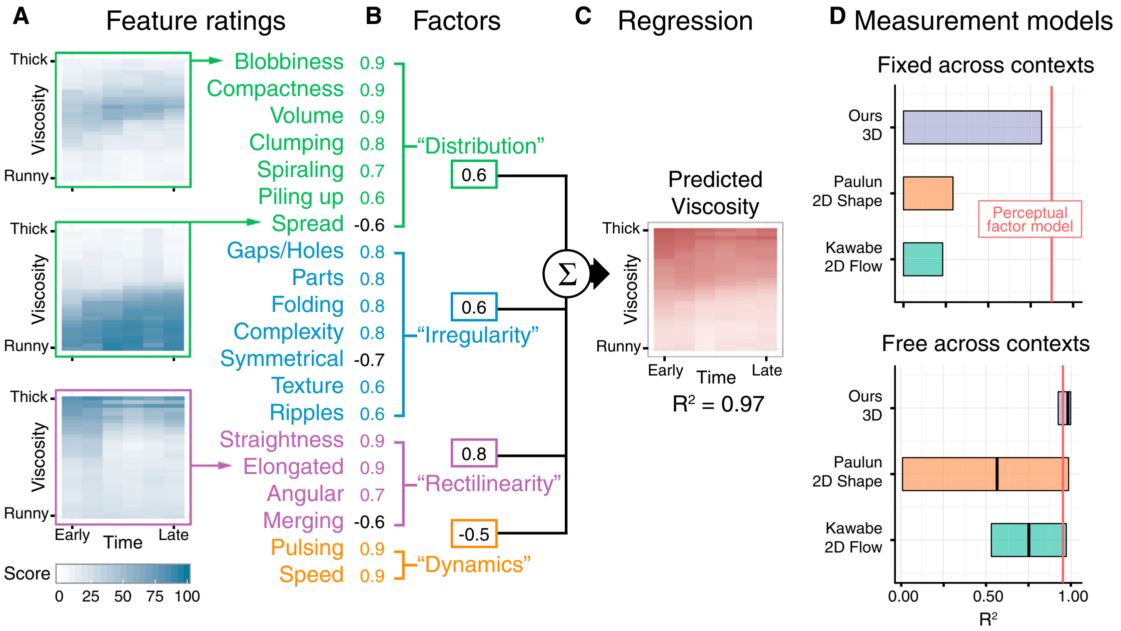

Figure 3. Model Creation

(A) Example ratings for three features from Experiment 3.

(B) Factor analysis weights for the 20 perceptual features into the corresponding four factors for which they have the largest weights.

(C) Multiple linear regression combines the four factors into a viscosity prediction for the data from Experiment 1.

(D) Comparison of three measurements-based models and the perceptual-factor model (red line). Regressions were performed on eight-scene stimulus set. Free and fixed across contexts refers to one set of weights for all scenes versus a separate set of weights for each scene. Free across contexts shows the lowest and the highest scene performance where the thick line is the mean across scenes.

See also Figure S4.

fast they move, how much they spread out or clump together, how irregular they are, and how rectilinear they are—determine the perception of viscosity across a very wide range of contexts.

To test the robustness of these conclusions, we also ran the factor analysis and regression in reverse, using the data from the eight scenes (Experiments 2 and 4) to build a model for predicting perceived viscosity. As before, this model predicts its training data very well (R^2 = 0.96, F(4,51) = 294, p < 0.001). When used to predict the viscosity ratings from the pouring scene (Experiment 1), this model also generalizes well (R^2 = 0.77, F(1,190) = 637, p < 0.001; again with no free parameters), although not as well as the original model, which is unsurprising given that only about a third as much training data was available (56 rather than 192 data points). To quantify the similarities between the two models, we computed representational dissimilarity matrices (RDM, [18]) describing the differences between stimuli in their respective factor spaces (Figure S3C). The RDMs correlated highly for both Experiment 3 (pouring scenes: R^2 = 0.65, F(1,18334) = 33,470, p < 0.001) and Experiment 4 (eight scenes: R^2 = 0.58, F(1,1538) = 2,090, p < 0.001), suggesting that the models learned similar representations of the stimuli from the feature ratings. Together these findings further suggest that representing stimuli using multiple complementary factors enables viscosity constancy.

Of course, some caution is required in interpreting these results. Although the range of liquid behavior we tested was broad, there may be some conditions where other, untested features could predict viscosity perception even better. Indeed, while these factors account for viscosity perception once a stimulus is identified as a liquid, it is highly unlikely they suffice for determining whether the stimulus is a liquid in the first place. Many non-liquid forms could appear as distributed, irregular, rectilinear, and dynamic as one of our stimuli without appearing to be a liquid of a specific viscosity. Thus, although these factors are important for viscosity estimation, they do not explain all aspects of liquid perception across all possible conditions. Nevertheless, the broader conclusion is that the visual system can achieve a high degree of constancy by representing liquids in a feature space incorporating multiple, complementary measurements. A similar approach has been proposed to account for errors of gloss perception [19, 20]; our results suggest that such an approach predicts both successes and failures of constancy in material perception more generally.

Why do these features work? The key challenge of constancy is that movies of the same liquid in different scenes are very different from one another in the image domain, while movies of different liquids in the same scene are much more similar (Figure 4A). Somehow the visual system must remap the representational space to organize the stimuli by their viscosity. We find that this is exactly what the midlevel features achieve. To investigate this, we performed principal component analysis (PCA) on the data from the second stimulus set (eight scenes). Figure 4A depicts each stimulus in the pixel similarity space by performing PCA on the rescaled grayscale pixel data of the entire video sequence. This represents the raw input to the visual system. The ellipses show standard errors around the mean for the seven

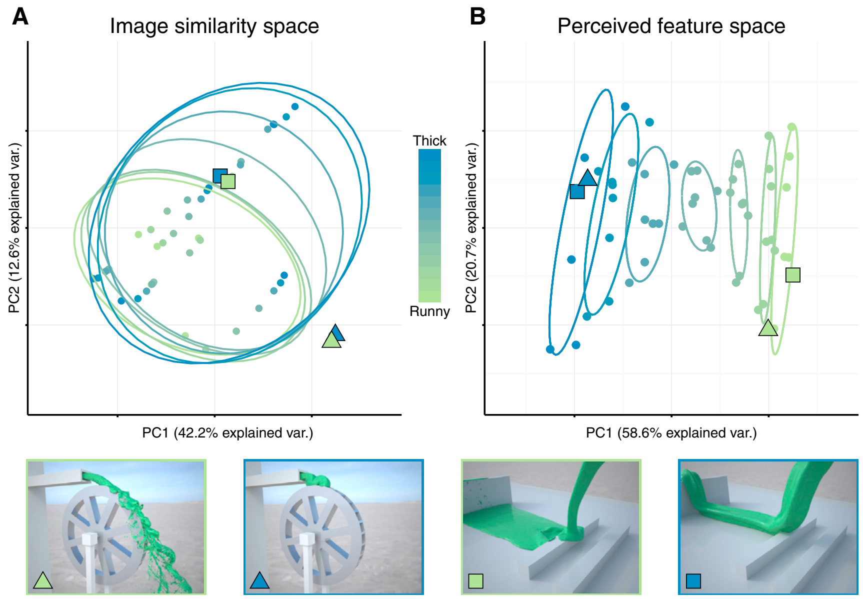

Figure 4. PCA Feature Space

(A) 2D PCA space derived from stimulus pixel values (36 million dimensions), a measurement of image similarity, representing the raw input to the visual system.

(B) 2D PCA space of the perceived shape features. Changes in viscosity align almost perfectly with the first component (R^2 = 0.96). Triangle and square data points indicate most runny (green) and viscous (blue) stimuli of two scenes. In image similarity space, the two viscosities are very similar and grouped together; in perceived feature space, the stimuli are separated by viscosity. This demonstrates that while the retinal input is dominated by extrinsic scene factors, the perceptual feature space unravels the common quantity across these scenes: the liquids' viscosity.

viscosities. The substantial overlap of the ellipses indicates that raw retinal image similarities provide a poor basis for viscosity perception, demonstrating the extent of the challenge confronting the visual system. In contrast, Figure 4B shows the PCA space of the features ratings and reveals a clear and systematic ordering of the stimuli by viscosity. It is important to emphasize that observers were simply instructed to rate different shape and motion features—viscosity was never mentioned. Despite this, the first principal component of the ratings correlates strongly with the actual viscosity (R^2 = 0.96, $F(1,54)$ = 1,212, $p < 0.001$). This demonstrates that despite massive physical variations across scenes, observers unknowingly arranged the stimuli according to the one common factor across these scenes: the viscosity. This impressive ability strongly suggests that the visual system achieves constancy by identifying features that transform the perceptual space to extract invariant material properties and negate the effects of other scene variables.

An important current debate in material perception research is the extent to which 2D image quantities are sufficient for material judgments [21, 22] or whether 3D surface structure plays a crucial role [23–25]. To provide some perspective on this, we developed a model using four shape metrics computed directly from the liquids' 3D meshes (see Figure S4 and STAR Methods), which we compared against two previous models, based on 2D optical flow [15] and 2D shape [14] (Figure 3D). The 2D models generalize poorly across our stimuli (Kawabe et al.: R^2 = 0.23; Paulun et al.: R^2 = 0.29). Only the novel 3D model predicts viscosity perception moderately well with fixed weights across scenes (R^2 = 0.81). Unsurprisingly, when we perform separate regressions for each scene independently (free weights), all three measurement models perform somewhat better. However, the results suggest that 3D representations contribute to the robustness of viscosity perception beyond simple 2D image measurements. How might 3D information be used? Simply representing local 3D structure at every surface point would be insufficient. Some degree of perceptual organization is required to group and summarize raw 3D measurements into quantities that relate to viscosity. We suggest that the midlevel perceptual factors pool and organize local 3D estimates to create robust viscosity cues.

Together, these results indicate that despite the extremely complex physics underlying fluid flow, we can predict viscosity perception using a small number of quite simple midlevel cues. A fascinating open question is how the visual system identifies which stimuli are liquids in the first place and then extracts information from features that robustly generalize across an even wider range of contexts than tested here (e.g., when spatial scale or the liquid's density also vary). In the long run, models of viscosity perception should be combined with a "front-end" that allows predicting viscosity perception directly from image sequences, presumably via 3D estimates. Such a model should also seek to predict the effects of lighting and the liquid's optical properties on perceived viscosity, although these effects are generally small [16].

One approach to liquid detection and feature selection would be through sophisticated—potentially innate [26–30]—physics-like internal models that capture the typical behavior of fluids. For example, Battaglia and colleagues suggest [31] that the visual system predicts liquids' future states through simulations based on internal models. They show that humans far outperform simple heuristics at predicting where and how liquids flow. Such "intuitive physics" approaches could potentially account for the successes of viscosity constancy we observe in our experiments: an internal model could be fit to liquids in a wide variety of different poses and contexts. Nevertheless, a challenge for any type of model is to predict the partial failures of viscosity constancy that we observe (e.g., the differences between the columns in Figure 2B).

An alternative approach would be to learn features from observation using large quantities of training data (e.g., learning of optimal speed or disparity encoding from natural scene data [32, 33]). Given the recent success of convolutional neural networks (CNNs) in predicting visual object recognition and its neural correlates [34–37], the visual system could likely learn to recognize liquids—and features diagnostic of viscosity—from sufficient training data. An interesting topic for future investigation is whether similar features emerge for both liquid detection

and viscosity estimation. Indeed, it would be fascinating to test whether CNNs arrive at similar features to the ones we identify here. Nevertheless, as such algorithms acquire their features through supervised training, a major challenge for their use as models of human perception is to explain where the training labels come from during human learning. We might associate certain ranges of viscosity with different liquids but are not explicitly taught a fine viscosity scale that relates across materials, and yet, as we find here, we eventually become surprisingly good and precise at identifying them across a wide range of conditions.

STAR★METHODS

Detailed methods are provided in the online version of this paper and include the following:

- KEY RESOURCES TABLE
- CONTACT FOR REAGENT AND RESOURCE SHARING
- EXPERIMENTAL MODEL AND SUBJECT DETAILS
- METHOD DETAILS
  - Stimuli
  - Procedure
  - Measurement models
- QUANTIFICATION AND STATISTICAL ANALYSIS
  - Factor analysis
  - Representational Similarity Analysis (RSA/RDMs)
  - Principal Component Analysis
- DATA AND SOFTWARE AVAILABILITY

SUPPLEMENTAL INFORMATION

Supplemental Information includes four figures, one table, and two movies and can be found with this article online at https://doi.org/10.1016/j.cub.2017.12.037.

ACKNOWLEDGMENTS

We thank NextLimit Technologies for software and support, as well as Karl Gegenfurtner, Niko Troje, and Flip Phillips for useful feedback. This work was funded by the DFG-funded Collaborative Research Center on "Cardinal Mechanisms of Perception" (SFB-TRR-135), an ERC Consolidator Award (ERC-2015-CoG-682859: "SHAPE"), and a Google Faculty Research Award.

AUTHOR CONTRIBUTIONS

J.J.R.v.A. generated stimuli, designed experiments, acquired data, developed mesh measurements, performed analysis and interpretation of data, and wrote manuscript. P.B. helped with mesh measurements, data interpretation, and manuscript editing. R.W.F. designed experiments, helped with mesh measurements and data interpretation, and co-wrote manuscript.

DECLARATION OF INTERESTS

The authors declare no competing interests.

Received: July 12, 2017
Revised: October 4, 2017
Accepted: December 19, 2017
Published: January 25, 2018

REFERENCES

1. Kraft, J.M., and Brainard, D.H. (1999). Mechanisms of color constancy under nearly natural viewing. Proc. Natl. Acad. Sci. USA *96*, 307–312.
2. Foster, D.H. (2011). Color constancy. Vision Res. *51*, 674–700.
3. Maloney, L.T., and Wandell, B.A. (1986). Color constancy: a method for recovering surface spectral reflectance. J. Opt. Soc. Am. A *3*, 29–33.
4. Motoyoshi, I., and Matoba, H. (2012). Variability in constancy of the perceived surface reflectance across different illumination statistics. Vision Res. *53*, 30–39.
5. Anderson, B.L. (2011). Visual perception of materials and surfaces. Curr. Biol. *21*, R978–R983.
6. Tarr, M.J., Williams, P., Hayward, W.G., and Gauthier, I. (1998). Three-dimensional object recognition is viewpoint dependent. Nat. Neurosci. *1*, 275–277.
7. Bülthoff, H.H., Edelman, S.Y., and Tarr, M.J. (1995). How are three-dimensional objects represented in the brain? Cereb. Cortex *5*, 247–260.
8. Rust, N.C., and Stocker, A.A. (2010). Ambiguity and invariance: two fundamental challenges for visual processing. Curr. Opin. Neurobiol. *20*, 382–388.
9. Biederman, I., and Gerhardstein, P.C. (1993). Recognizing depth-rotated objects: evidence and conditions for three-dimensional viewpoint invariance. J. Exp. Psychol. Hum. Percept. Perform. *19*, 1162–1182.
10. Riesenhuber, M., and Poggio, T. (2000). Models of object recognition. Nat. Neurosci. *3* (*Suppl*), 1199–1204.
11. Gilchrist, A., Kossyfidis, C., Bonato, F., Agostini, T., Cataliotti, J., Li, X., Spehar, B., Annan, V., and Economou, E. (1999). An anchoring theory of lightness perception. Psychol. Rev. *106*, 795–834.
12. Adelson, E.H. (2000). Lightness Perception and Lightness Illusions. In the New Cognitive Neurosciences, M. Gazzaniga, ed. (MIT Press), pp. 339–351.
13. Marlow, P.J., Kim, J., and Anderson, B.L. (2012). The perception and misperception of specular surface reflectance. Curr. Biol. *22*, 1909–1913.
14. Paulun, V.C., Kawabe, T., Nishida, S., and Fleming, R.W. (2015). Seeing liquids from static snapshots. Vision Res. *115* (Pt B), 163–174.
15. Kawabe, T., Maruya, K., Fleming, R.W., and Nishida, S. (2015). Seeing liquids from visual motion. Vision Res. *109*, 125–138.
16. van Assen, J.J.R., and Fleming, R.W. (2016). Influence of optical material properties on the perception of liquids. J. Vis. *16*, 12.
17. Horn, J.L. (1965). A rationale and test for the number of factors in factor analysis. Psychometrika *30*, 179–185.
18. Kriegeskorte, N., Mur, M., and Bandettini, P. (2008). Representational similarity analysis - connecting the branches of systems neuroscience. Front. Syst. Neurosci. *2*, https://doi.org/10.3389/neuro.06.004.2008.
19. Kim, J., Marlow, P.J., and Anderson, B.L. (2012). The dark side of gloss. Nat. Neurosci. *15*, 1590–1595.
20. Fleming, R.W. (2012). Human perception: Visual heuristics in the perception of glossiness. Curr. Biol. *22*, R865–R866.
21. Motoyoshi, I., Nishida, S., Sharan, L., and Adelson, E.H. (2007). Image statistics and the perception of surface qualities. Nature *447*, 206–209.
22. Sharan, L., Liu, C., Rosenholtz, R., and Adelson, E.H. (2013). Recognizing materials using perceptually inspired features. Int. J. Comput. Vis. *103*, 348–371.
23. Marlow, P.J., Todorović, D., and Anderson, B.L. (2015). Coupled computations of three-dimensional shape and material. Curr. Biol. *25*, R221–R222.
24. Marlow, P.J., and Anderson, B.L. (2015). Material properties derived from three-dimensional shape representations. Vision Res. *115* (Pt B), 199–208.
25. Marlow, P.J., Kim, J., and Anderson, B.L. (2017). Perception and misperception of surface opacity. Proc Natl Acad Sci USA *114*, 13840–13845.
26. Battaglia, P.W., Hamrick, J.B., and Tenenbaum, J.B. (2013). Simulation as an engine of physical scene understanding. Proc. Natl. Acad. Sci. USA *110*, 18327–18332.
27. Rips, L.J., and Hespos, S.J. (2015). Divisions of the physical world: Concepts of objects and substances. Psychol. Bull. *141*, 786–811.

28. Hespos, S.J., and vanMarle, K. (2012). Physics for infants: characterizing the origins of knowledge about objects, substances, and number. Wiley Interdiscip. Rev. Cogn. Sci. *3*, 19–27.
29. Hespos, S.J., Ferry, A.L., and Rips, L.J. (2009). Five-month-old infants have different expectations for solids and liquids. Psychol. Sci. *20*, 603–611.
30. Spelke, E. (1994). Initial knowledge: six suggestions. Cognition *50*, 431–445.
31. Bates, C.J., Yildirim, I., Tenenbaum, J.B., and Battaglia, P.W. (2015). Humans predict liquid dynamics using probabilistic simulation. Proceedings of the 37th Annual Conference of the Cognitive Science Society.
32. Burge, J., and Geisler, W.S. (2014). Optimal disparity estimation in natural stereo images. J. Vis. *14*, 1.
33. Burge, J., and Geisler, W.S. (2015). Optimal speed estimation in natural image movies predicts human performance. Nat. Commun. *6*, https://doi.org/10.1038/ncomms8900.
34. Khaligh-Razavi, S.M., and Kriegeskorte, N. (2014). Deep supervised, but not unsupervised, models may explain IT cortical representation. PLoS Comput. Biol. *10*, e1003915.
35. Yamins, D.L., Hong, H., Cadieu, C.F., Solomon, E.A., Seibert, D., and DiCarlo, J.J. (2014). Performance-optimized hierarchical models predict neural responses in higher visual cortex. Proc. Natl. Acad. Sci. USA *111*, 8619–8624.
36. Güçlü, U., and van Gerven, M.A. (2015). Deep neural networks reveal a gradient in the complexity of neural representations across the ventral stream. J. Neurosci. *35*, 10005–10014.
37. Cichy, R.M., Khosla, A., Pantazis, D., Torralba, A., and Oliva, A. (2016). Comparison of deep neural networks to spatio-temporal cortical dynamics of human visual object recognition reveals hierarchical correspondence. Sci. Rep. *6*, 27755.
38. Brainard, D.H. (1997). The psychophysics toolbox. Spat. Vis. *10*, 433–436.
39. Pelli, D.G. (1997). The VideoToolbox software for visual psychophysics: transforming numbers into movies. Spat. Vis. *10*, 437–442.
40. Bridson, R. (2015). Fluid simulation for computer graphics (CRC Press).
41. Bouguet, J.-Y. (2000). Pyramidal implementation of the Lucas Kanade feature tracker description of the algorithm. Intel Corporation, Microprocessor Research Labs. OpenCV Documents.
42. Koenderink, J.J., and van Doorn, A.J. (1992). Surface shape and curvature scales. Image Vis. Comput. *10*, 557–564.

STAR★METHODS

KEY RESOURCES TABLE

REAGENT or RESOURCE	SOURCE	IDENTIFIER
Deposited Data		
Raw data	This paper	http://doi.org/10.5281/zenodo.1136202
Stimuli	This paper	http://doi.org/10.5281/zenodo.1136202
Software and Algorithms		
RealFlow 2014/2015 (V. 8.1.2.0192/V. 9.1.2.0193)	NextLimit Technologies	http://www.nextlimit.com/realflow/
Maxwell Render (V. 3.0.1.3)	NextLimit Technologies	http://www.nextlimit.com/maxwell/
MATLAB	MathWorks	https://www.mathworks.com/products/matlab.html; RRID:SCR_001622
Psychophysics Toolbox	[38, 39]	http://psychtoolbox.org/; RRID:SCR_002881
R	R Foundation	https://www.r-project.org; RRID:SCR_001905
R analysis code	This paper	http://doi.org/10.5281/zenodo.1136202

CONTACT FOR REAGENT AND RESOURCE SHARING

Further information and requests for resources should be directed to and will be fulfilled by the Lead Contact, Jan Jaap van Assen (mail@janjaap.info).

EXPERIMENTAL MODEL AND SUBJECT DETAILS

Groups of twelve observers rated perceived viscosity in the first two experiments. In Experiments 3 and 4, where shape features were rated, two separate groups were formed. Each group rated only ten of the twenty shape features. In Experiment 3, twelve observers participated in each group, and in Experiment 4, ten observers participated in each group. In total, over all four experiments, 68 observers participated. The average observer age was 25.0 (SD = 4.82). 45 observers were female and 23 male. In the control experiments a total of 21 observers participated (4 in the brainstorming experiment, 8 in the semantic (word-list) matching experiment, and 9 in the factor rating experiment). The average age was 24.3 (SD = 3.89), 14 observers were female and 7 male. All observers gave written consent prior to the experiment and were paid for participating. All observers reported having normal or corrected-to-normal vision. Experiments were conducted in accordance with the Declaration of Helsinki and prior approval was obtained from the local ethics committee of Giessen University.

METHOD DETAILS

Stimuli

Two stimulus sets were used in the four experiments. Set 1 consisted of a pouring liquid scene that was simulated with 32 viscosity steps. Each 10 s long animation was divided into six time periods of 1.67 s each for Experiments 1 and 3. This resulted in a total of 192 stimuli (32 viscosities × 6 time periods). Set 2 consisted of eight different scenes each simulated with seven viscosity steps. The duration of each stimulus in Set 2 was the full 10 s because (1) In Experiment 1 we found that time had very little effect on perceived viscosity, and (2) due to the very wide range of speeds across scenes, there were long time periods for some scenes with viscous liquids, where the liquid had not yet entered the scene, which obviously would have made viscosity estimation impossible. Thus, Set 2 contained 56 stimuli (7 viscosities × 8 different scenes).

Simulation

Stimuli were generated using RealFlow 2014/2015 (V. 8.1.2.0192/V. 9.1.2.0193; NextLimit Technologies, Madrid, Spain). The pouring liquid scene (Experiments 1 and 3) consisted of 32 different viscosities ranging from 0.001 to 80.30 Pa·s. In Experiments 2 and 4, seven different viscosities were tested ranging from 0.004 Pa·s to 7.74 Pa·s in eight different scenes. Viscosity values were selected from a logarithmically spaced scale of 64-steps between 0.001 Pa·s and 100 Pa·s (equivalent from water to molten glass). RealFlow provides multiple particle solvers; in this case the "Hybrido" particle solver was used, making it possible to specify the *dynamic viscosity* of the liquids in real physical units (Pa·s). Hybrido is a FLIP (Fluid-Implicit Particle) solver using a hybrid grid and particle technique to compute a numerical solution to the Navier-Stokes equations describing viscous fluid flow [40]. Discrete particles carry all information for the fluid simulation, but the solution to the equations is carried out on a grid. Once the grid solve is complete, the

particles gather the information required from the grid to move forward in time to the next frame. Finally, a meshing algorithm uses the particles to calculate the fluid boundary. When visible artifacts occur, it is mostly due to the mesh calculation, not the underlying physics solver. The density of the liquids was held constant at one kilogram per liter. The number of particles varied across scenes, with a maximum of roughly 5 million particles. All scenes were simulated in a space of roughly one cubic meter. Gravity was the main external force acting on the liquid, however in some cases an additional noise force field was used to achieve better scene-liquid interaction. The simulated animations had a total duration of ten seconds (300 frames at 30fps).

Rendering

The render engine used to generate the final image frames was Maxwell (V. 3.0.1.3; NextLimit Technologies, Madrid, Spain). Images were rendered at 800 × 600 resolution and the scene was lighted using an HDR light probe depicting a beach scene (from the Maxwell Resource Library by Dosch Design). The liquid of the pouring scene (Experiments 1 and 3) was rendered with a translucent material. The liquid in all other scenes was of a green opaque material. Previous research has shown that optical material appearance of liquids barely influences viscosity judgements [16].

Procedure

Experiment 1 and 2: Rating viscosity

The experiments were performed on a Dell T3500 with a Dell U2412M 24-inch monitor using factory default settings, gamma of 2.2 and a resolution of 1920 × 1200 pixels. MATLAB 2015a (v. 8.5.0.197613) and the Psychtoolbox library (v. 3.0.12) [38, 39] were used to run the experiments. Observers completed a short training session before starting the experiment. The training consisted of a single trial in which the maxima and minima of the stimuli were presented and the observer could get acquainted with the interface. For Experiments 2 and 4, all eight scenes were shown as well. During each trial, eight stimuli were shown with a rating bar transparently projected over each stimulus (Movie S2). There was no time limit and once all rating bars were set the observer could continue to the next trial. Corrections during the trial were possible and the observer was free to choose in which order the stimuli were rated. Each stimulus was repeated four times during the experiment but the position and combinations with other stimuli were chosen randomly for each trial.

Experiment 3 and 4: Rating shape features

The setup was the same as in Experiments 1 and 2. Experiments 3 and 4 were divided into two groups of observers, each rating ten of the twenty shape features. The stimuli were organized by viscosity on the screen. This was done to make it easier to rate the shape features. In the case of Experiment 3, 32 stimuli of the same time period were shown simultaneously. For Experiment 4, seven stimuli of one scene were shown. There were no repetitions in Experiment 3 and in Experiment 4 every trial was shown twice, in random order. Each shape feature name was presented in the top left of the screen and an additional description was provided for clarity. All experiments were performed in German and have been translated to English for presentation here, see table S1 for a full list of shape features and descriptions.

Control experiment 1: Brainstorming new word list

We asked four observers to brainstorm ‘shape features’ while viewing videos of the pouring liquids (full 10 s duration). There was a short training stage in which we explained the concept of shape features with examples using cars and plants. We carefully used examples that would not overlap with features in liquids. Individually, each observer wrote down as many shape features as possible, after which the four observers were instructed to work together to pick the most descriptive twenty features. This closely resembles the way we selected the features ourselves.

Control experiment 2: Semantic matching of word lists

In this experiment, eight observers were asked to rate the similarity between our original word list (A) and new words generated in control experiment 1 (B). The videos of the pouring liquids were shown to provide some context. For each word in one list, the observer had to select similar words from the other word list. Observers were not required to choose similar words if there were none, and a maximum of three similar words for each item was allowed. The similarity of each of the matching words was then rated as ‘high similarity’, ‘intermediate similarity’, and ‘little similarity’. This experiment was performed in both directions, so wordlist A was matched with wordlist B and vice versa. This enabled us to judge the similarity between the two word lists.

Control experiment 3: Factor ratings

In this control experiment we asked nine observers to rate the four factors (Distribution, Irregularity, Rectilinearity and Dynamics) directly, instead of the 20 features. Apart from this, the experimental procedure was the identical to the main Experiment 3 in which the 20 features were rated.

Measurement models

2D Motion flow model

Kawabe et al. [15] showed that the mean speed of optical flow is highly predictive of perceived viscosity. To evaluate whether motion cues are able to predict viscosity in our stimuli, we used the same iterated pyramidal Lucas-Kanade method [41] to calculate optical flow. We found that flow speed correlated poorly with perceived viscosity in the pouring liquids scene (R^2 = 0.01, F(1,190) = 2.297,

p = 0.13). There are at least two possible reasons for this: first, the liquid was translucent, which could hinder optical flow computations; second, the second half of the movie does not contain much motion across the entire viscosity range. Optical flow for the other eight different scenes and opaque liquids also did not perform very well (R^2 = 0.23, F(1,54) = 16.24, p < .01). Only when we perform regression analysis for each specific scene do we see that for some specific scenes optical flow is a good predictor, with an average of R^2 = 0.75, and minima an maxima between R^2 = 0.53 and R^2 = 0.97. The large variations in performance of the motion predictor suggest that the visual system likely uses other cues in addition to speed to infer viscosity.

2D Image statistics model

Paulun et al. [14] found that twenty simple 2D shape statistics derived from the liquids' silhouette predict perceived viscosity surprisingly well. The statistics include measurements of shape, area, curvature, spatial distribution and perimeter, among others. We applied the same measurements to our stimuli, having excluded frames where there was not enough liquid (fewer than 300 pixels, i.e, < 0.06% of image) and areas with only one-pixel width (to avoid errors in the contour measurements). Paulun et al. did not apply a regression but simply took the mean of the normalized measurements. Without fitting they found the model predicted perception in their stimuli extremely well (r = 0.99, p < 0.001). We applied the model to the second stimulus set (eight scenes) and found a much poorer fit (R^2 = 0.29, F(1,54) = 22.27, p < 0.001). Like Paulun et al. we used only a single predictor, the mean of all normalized measurements across our eight scenes. Performing a regression for each scene independently yield highly variable performance, ranging from R^2 = 0.01 to R^2 = 0.99. This shows that in some cases simple 2D shape measurements are sufficient to predict viscosity very well. However such cues are not flexible or invariant enough to achieve similar performance across contexts. Generalizing the model to use all 20 features as separate predictors in a regression (rather than the mean across measurements) yields R^2 = 0.80, F(20,35) = 7.19, p < 0.001, compared to R^2 = 0.81, F(4,51) = 55.88, p < 0.001 for our 3D model with only four predictors. The difference in performance is likely due to the fact that the four 3D measurements generalize better across scenes and contain less covariance than the twenty 2D measurements.

3D Shape measurements model

One advantage of computer-simulated liquids is the generation of detailed 3D meshes of the liquids. From these, we derived four 3D measurements (Figure S4) that were loosely inspired by some of the perceptual features in the regression model. Specifically, (1) mean absolute curvature weighted by the shape index [42], which emphasizes angular features, (2) the sum of absolute vertical normal coordinates, which captures the tendency of liquids to form horizontal planes as they spread out, (3) the vertical position of the center of mass, which tends to be higher when the liquid piles up, and (4) total absolute curvature of the liquid, which tends to be large when the surface has many local convolutions. As the pouring liquids sequence is divided into six periods, we compensated for large differences in mesh size over time by normalizing the median value of each feature over the different time periods. This was not necessary for the stimuli used in Experiment 2 and 4 where the entire 10 s time sequence was shown and we could simply take the average measurement value over 300 frames. We did apply normalization of each measurement across the scenes. To compare performance with the other models we applied a multiple linear regression on the second stimulus set with eight scenes. We find the 3D model performs much better than the other two (R^2 = 0.81, F(4,54) = 55.88, p < 0.001). When we apply the regression separately for each scene the mean is R^2 = 0.98 across scenes. It is important to note however, that this performance is achieved even though the mesh measurements do not correlate with the perceived feature ratings across contexts (mean R^2 = 0.04). This means that although a linear combination of the mesh measurements can explain perceived viscosity relatively well, there is no direct correspondence between these measurements and the features that our observers judged.

QUANTIFICATION AND STATISTICAL ANALYSIS

All experiments were performed in MATLAB using Psychtoolbox (v. 3.0.12) [38, 39]. All analyses were performed in R. The code is publicly available and can be downloaded here: http://doi.org/10.5281/zenodo.1136202. All dependencies of external packages used in R are clearly documented in the code. No observers were excluded from the analysis.

Factor analysis

We performed a maximum likelihood factor analysis using the R 'psych' package. To determine how many factors there were in the dataset, we applied Horn's parallel analysis [17]. We applied the Harman method to calculate the scores, applying the loadings to the actual data.

Representational Similarity Analysis (RSA/RDMs)

For a comprehensive description of Representational Similarity Analysis, we refer to [18]. The representational dissimilarity matrices (RDMs) in figure S3C were calculated using the Euclidean distances between observations in the 4D factor space, with each dimension representing one of the factors. The linear regression used to quantify similarity between RDMs was performed on the lower triangles of the two matrices (i.e., diagonal and upper triangle excluded from analysis).

Principal Component Analysis

To perform PCA on the raw image similarity space (Figure 4A), we halved the images to 400 × 300 pixels, and converted the images to grayscale using the following conversion values (0.2989 * R + 0.5870 * G + 0.1140 * B). The resulting dataset contains 36 million

dimensions for each of the 56 stimuli (i.e., over 2 billion observations in total). We include this PCA data as a separate, comma separated file.

DATA AND SOFTWARE AVAILABILITY

All data, analysis code, and stimuli are available on Zenodo at http://doi.org/10.5281/zenodo.1136202. Any questions should be directed to the Lead Contact (mail@janjaap.info).

Current Biology, Volume *28*

Supplemental Information

Visual Features in the Perception of Liquids

Jan Jaap R. van Assen, Pascal Barla, and Roland W. Fleming

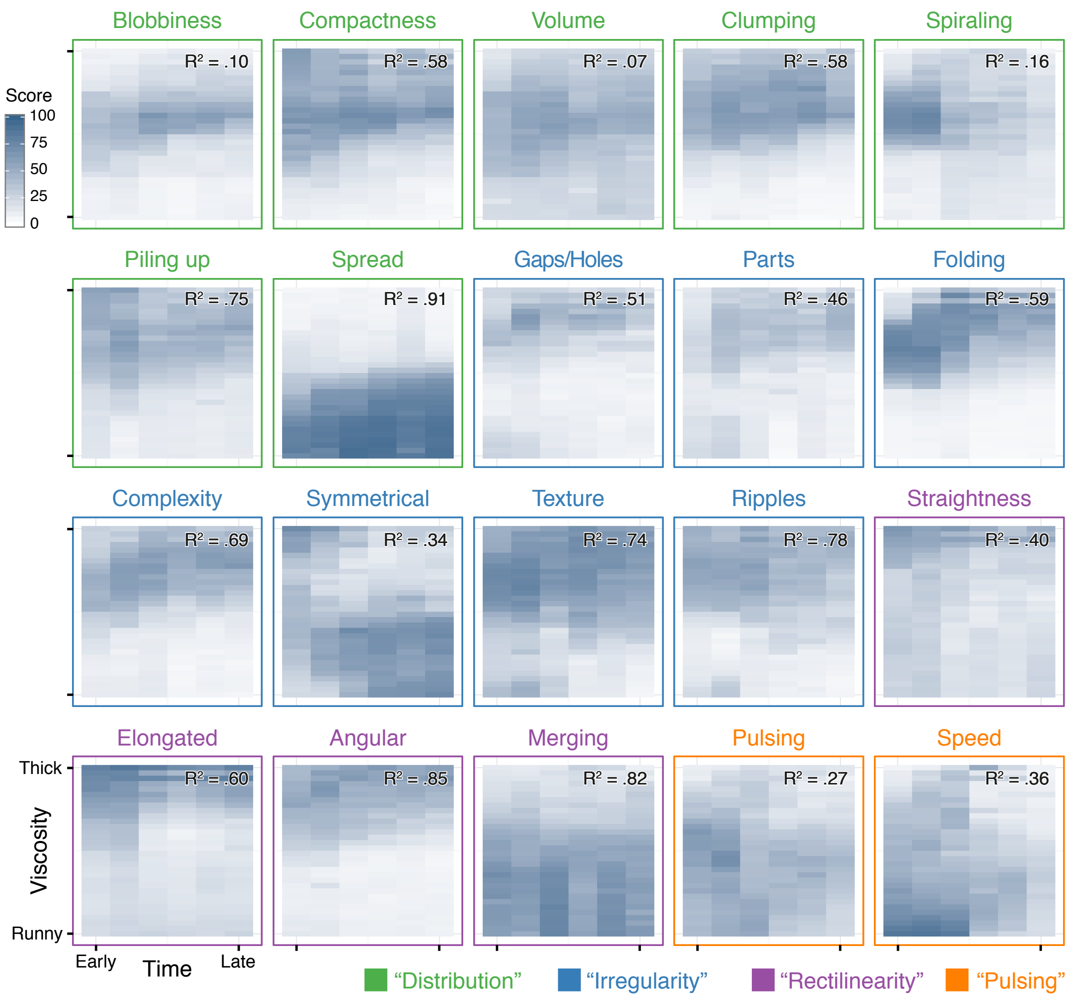

Figure S1: Experiment 3 feature ratings. Related to Figure 1: Mean midlevel feature ratings of Experiment 3 for all twenty shape features, colour coded by the factors for which they have the largest weights. Y-axis: viscosity (32 viscosities, from runny 0.001 Pa·s to thick 80.30 Pa·s); X-axis: time (six time periods of 1.67 seconds or 10 seconds divided by six). The feature judgments generally varied in complex (often non-monotonic) ways as a function of viscosity and time period.

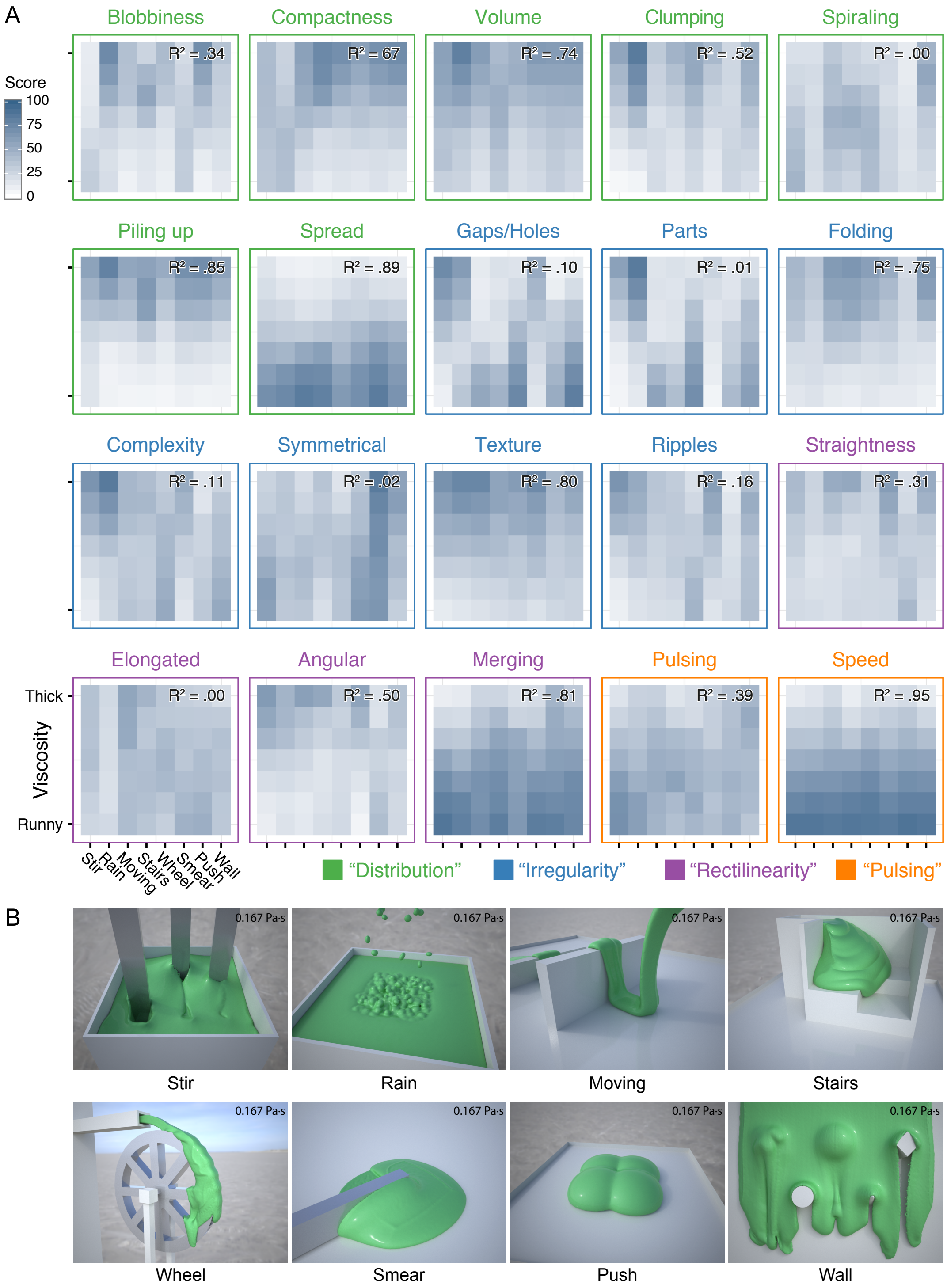

Figure S2: Experiment 4 feature ratings. Related to Figure 2: A) Mean midlevel feature ratings of Experiment 4 for all twenty shape features, colour coded by the factors for which they have the largest weights. Y-axis: viscosity (7 viscosities, from runny 0.004 Pa·s to thick 7.74 Pa·s); X-axis: eight different scenes. The feature judgements vary considerably on scene-by-scene basis, indicating that our simulated scenes capture a wide range of different liquid behaviours. B) The eight scenes with an intermediate viscosity (0.167 Pa·s).

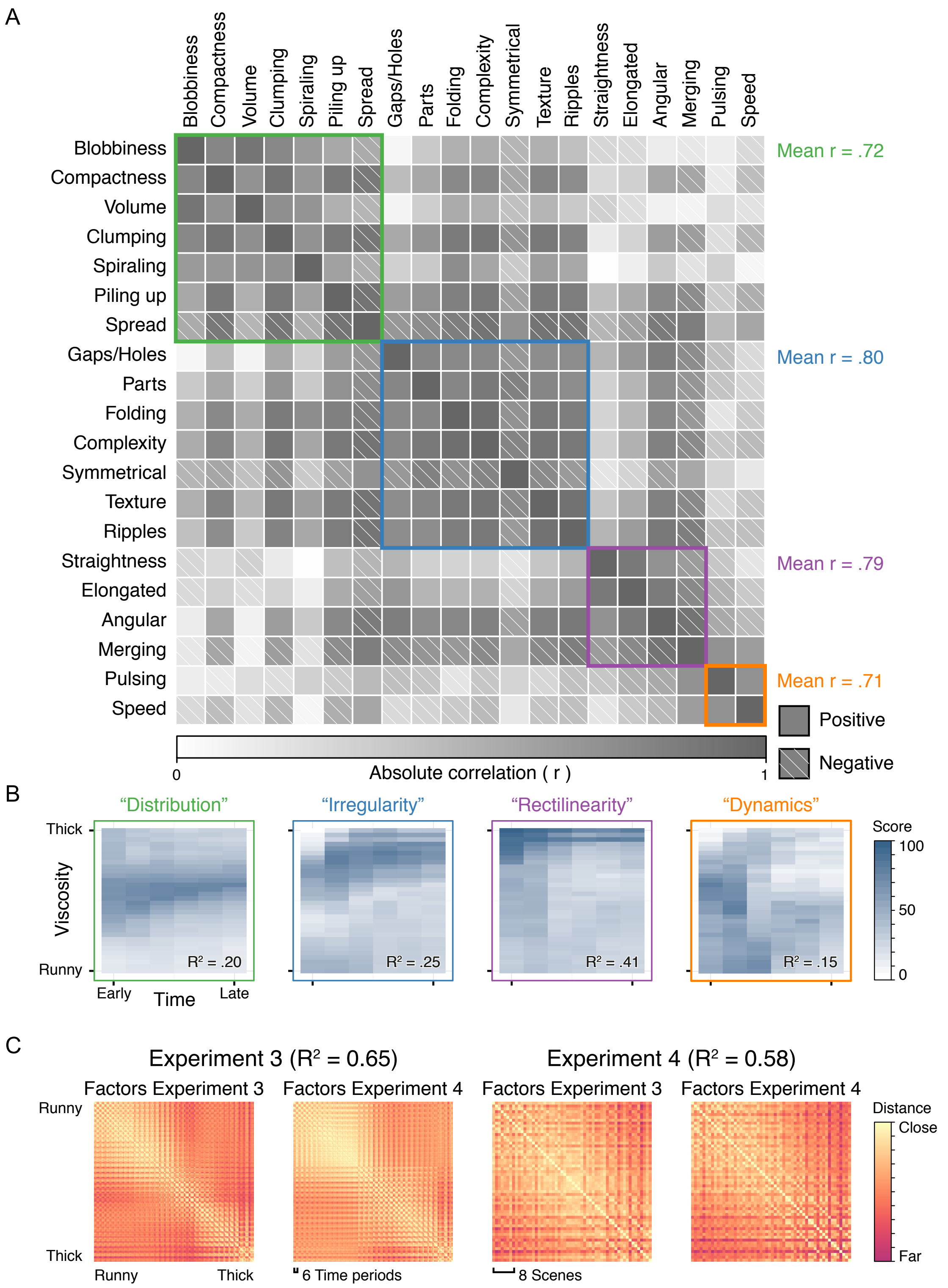

Figure S3: Factor analysis. Related to Figure 3: A) Correlations between perceptual feature ratings from Experiment 3; gray shade indicates absolute magnitude of correlation, stripes indicate negative correlations. Coloured frames indicate the four factors; *r*-values indicate mean absolute correlations between features within each factor, the overall mean absolute correlation is $r = 0.57$. B) The four factors that result from applying the factor loadings to the twenty perceptual features. R^2 values indicate linear regression between each

factor on its own and the viscosity ratings from Experiment 1. The complementary nature of the factors means that each on its own predicts only a small proportion of the variance, but combined in a multiple linear regression, they explain 97% of the variance in the viscosity ratings. C) Representational Dissimilarity Matrices (RDMs) for the two regression models, derived from the Pouring scene (left) and the 8 scenes (right). In each case we apply factors derived from Experiment 3 and Experiment 4 and quantify how similar these factor spaces are. Colours represent the Euclidean distance between the corresponding pair of stimuli in the respective factor-space representation (red: dissimilar, yellow: similar). The R^2-score indicates the explained variance between the lower triangles of the two matrices (i.e., diagonal and upper triangle excluded from analysis).

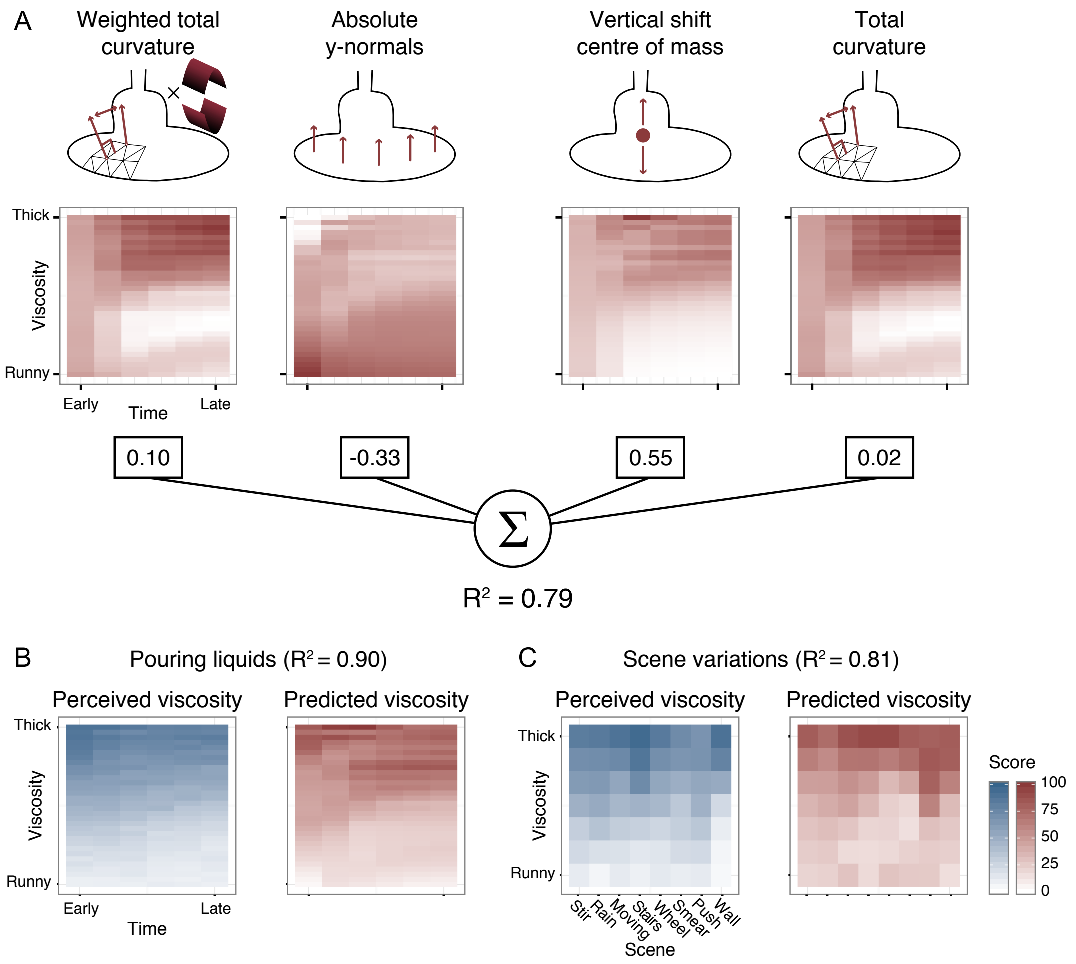

Figure S4: 3D measurements. Related to Figure 3D: A) Schematics of the four mesh measurements used in the 3D stimulus-computable model, along with values for all stimuli in the pouring scene (Experiments 1 and 3). A regression model using the weights below each mesh measurement predicts 79% of the variance in viscosity ratings across all scenes. B) Human viscosity ratings (blue) and mesh model predictions (mauve) for Experiment 1 (pouring liquids). R^2 score indicates regression for a model fit only to these data. C) Human viscosity ratings (blue) and mesh measurements predictions (mauve) for Experiment 3 (eight scenes). R^2 score indicates regression for a model fit only to these data.

Shape feature	Description
Symmetrical	How symmetrical the shape is
Compactness	How tightly arranged the shape is
Clumping	How much the shape features distinct clumps
Folding	How much does the shape fold back on itself
Pulsing	How much does shape change in a rhythmical repeating way
Merging	How the shape absorbs into itself
Speed	How quickly the shape changes or moves
Spiraling	How much does the shape change in a spiral movement or form
Elongated	How much is the shape stretched out in a single direction
Texture	How much does the surface have variations rather than being smooth
Blobbiness	How rounded or bulbous is the shape
Piling up	How piled up is the shape
Straightness	How much does the shape contain straight features
Complexity	How complex is the shape i.e. not simple
Spread	How spread out is the shape
Ripples	How much does the shape feature ripples
Gaps/Holes	How much does the shape shows gaps or holes
Parts	How much does the shape consist of multiple parts rather than one single part
Angular	How sharp or angular are the features of the shape
Volume	How voluminous is the shape

Table S1: Feature list. Related to Figure 1 and 2: First column showing the twenty different shape features used in Experiment 3 and 4. Second column showing the corresponding description given as additional information to make it easier to rate the corresponding feature (here translated from German). The separation in the middle of the table shows the two different feature groups, each observer only rated ten features out one of these two groups.
